# Curriculum-Guided Adversarial Learning for Enhanced Robustness in 3D Object Detection

**DOI:** 10.3390/s25061697

**Published:** 2025-03-09

**Authors:** Jinzhe Huang, Yiyuan Xie, Zhuang Chen, Ye Su

**Affiliations:** 1 College of Computer and Information Science, Chongqing Normal University, Chongqing 401331, China; 2022210516054@stu.cqnu.edu.cn; 2 College of Electronics and Information Engineering, Southwest University, Chongqing 400715, China; cxzplus@email.swu.edu.cn (Z.C.); suye12345@swu.edu.cn (Y.S.)

**Keywords:** 3D object detection, adversarial learning, LiDAR, PointPillars

## Abstract

The pursuit of robust 3D object detection has emerged as a critical focus within the realm of computer vision. This paper presents a curriculum-guided adversarial learning (CGAL) framework, which significantly enhances the adversarial robustness and detection accuracy of the LiDAR-based 3D object detector PointPillars. By employing adversarial learning with prior curriculum expertise, this framework effectively resists adversarial perturbations generated by a novel attack method, P-FGSM, on 3D point clouds. By masterfully constructing a nonlinear enhancement block (NEB) based on the radial basis function network for PointPillars to adapt to the CGAL, a novel 3D object detector named Pillar-RBFN was developed; it exhibits intrinsic adversarial robustness without undergoing adversarial training. In order to tackle the class imbalance issue within the KITTI dataset, a data augmentation technique has been designed that singly samples the point cloud with additional ground truth objects frame by frame (SFGTS), resulting in the creation of an adversarial version of the original KITTI dataset named Adv-KITTI. Moreover, to further alleviate this issue, an adaptive variant of focal loss was formulated, effectively directing the model’s attention to challenging objects during the training process. Extensive experiments demonstrate that the proposed CGAL achieves an improvement of 0.8∼2.5 percentage points in mean average precision (mAP) compared to conventional training methods, and the models trained with Adv-KITTI have shown an enhancement of at least 15 percentage points in mAP, compellingly testifying to the effectiveness of our method.

## 1. Introduction

**Figure 1:**
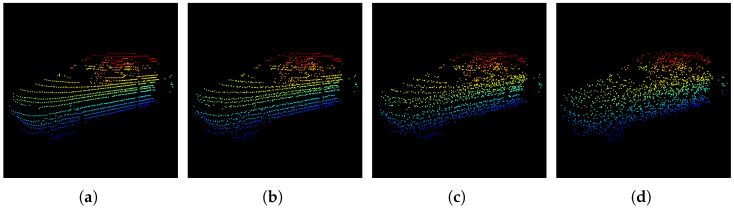
An illustration of a car point cloud object from the KITTI dataset [11] under different perturbation magnitudes, ϵ∈{0,1,5,10}. Diagram (**a**) corresponds to the original unperturbed version (ϵ=0), while diagrams (**b**), (**c**), and (**d**) represent perturbed versions corresponding to ϵ=1, ϵ=5, and ϵ=10, respectively. It can be observed that when the perturbation magnitude ϵ is 1 or 5, the contours of the car points remain relatively clear. Nevertheless, when ϵ equals 10, the points deviate from the original LiDAR scanning trajectory.

Recently, the 3D perception task has garnered notable attention based on various sensors such as LiDAR, radar, and camera, particularly deployed within autonomous driving and robotic applications [1,2]. Given the critical dependence of these systems on the precise detection of their surroundings, the stability of perceptual algorithms holds paramount importance and continues to pose a formidable challenge [3]. LiDAR-based 3D object detection serves as a typical 3D perception task that requires precisely locating the position of the object within the scene point cloud data generated by the LiDAR sensor [4]. Specifically, it entails the prediction of the 3D bounding box parameters of the objects such as sizes, coordinates, and confidence scores under the LiDAR coordinate system. However, current methods frequently overlook the implications of adversarial examples [5,6,7], which originate from the field of adversarial attacks on DeepNet, where specific methods are used to generate noise perturbations that are incorporated into clean data (the purpose of these perturbations is to cause DeepNet to induce erroneous predictions). Figure 1 visualizes the results of a point cloud vehicle object under varying extents of perturbations, where it can be discerned that the points still maintain their regular trajectories with minimal perturbations in diagrams (a) and (b). In contrast, the more acute perturbations in diagrams (c) and (d) introduce ambiguity into the point cloud, potentially confusing existing detectors. The vulnerability of deep neural network (DeepNet) models to adversarial perturbations poses a significant challenge, which may cause severe incidents [8] in reality, as even minor alterations to input data can yield incredibly erroneous predictions [9,10].

In real-world scenarios, several adverse weather conditions, such as rainy, foggy, and snowy days, as well as natural disasters, like locust plagues and sandstorms, or uncontrollable factors, such as jitters in laser emitters and communication interference during data transmission, can induce distortions in LiDAR point cloud densities and positions. Consequently, the resultant point cloud examples may be unrecognizable by the detection systems of autonomous vehicles, potentially triggering a series of safety incidents, such as collisions, abrupt braking, and erratic steering, highlighting the urgency of enhancing the adversarial robustness of 3D object detection models based on DeepNet [12,13]. Adversarial learning encompasses any process that integrates adversarial mechanisms to augment model performance, with adversarial training exemplifying a prototypical approach, which has emerged as a vital area of research aimed at improving DeepNet’s adversarial robustness by training algorithms to recognize and counteract adversarial examples, which are created by applying subtly crafted perturbations to the input data [14]. By implanting adversarial training, models obtain the ability to differentiate between benign and malicious inputs. In parallel, curriculum learning is a machine learning strategy that mimics the human learning process by breaking down tasks into a progressive sequence of phases [15]. This approach typically covers the initial application of comprehensible samples during the training phase, thereafter supplying increasingly arduous samples to equip the models’ effective learning. For instance, Ref. [16] draws upon the principles of curriculum learning to design its loss function, enabling the model to converge rapidly. Similarly, Ref. [17] employed a progressive training approach for its 3D point cloud recognition model based on a transformer module in an easy-to-hard manner, attaining more enhanced accuracy.

This article focuses on the adversarial robustness of a LiDAR-based 3D object detection model, PointPillars [5]. It initially constructs a nonlinear enhancement block (NEB) based on the radial basis function network (RBFN) and incorporates it into the PointPillars model [5]; thereafter, a detection architecture specifically tailored to the current task is manufactured, which is referred to as Pillar-RBFN. It was demonstrated that, even in the absence of adversarial training, Pillar-RBFN maintains a mean average precision (mAP) that exceeds at least 85% across various perturbation magnitudes, exhibiting more commendable adversarial robustness compared to its peers. Given that the majority of existing public point cloud datasets, such as KITTI [11], are collected under clear weather conditions, they typically exhibit a reduced level of sample noise. Consequently, in order to train the Pillar-RBFN adversarially, this study generates perturbations for point clouds to simulate scenarios with low signal-to-noise ratios found in real-world conditions, such as heavy rainfall weather. However, since the majority of existing perturbation generation methods predominantly focus on the field of image vision, there exists a gap in the literature concerning 3D point clouds [12]; thus, our method further proposes a novel technique, P-FGSM, to generate adversarial perturbations based on point clouds. Moreover, conventional adversarial training methods employ strong perturbations from the outset, such as projected gradient descent (PGD) [18], which may induce oscillations in the model’s optimization process. Consequently, our approach incorporates the principles of curriculum learning, facilitating a gradual progression in the training process. Furthermore, it dynamically adjusts the perturbation magnitude parameters to enable the model to adapt to varying levels of detection difficulty. By synthesizing the strengths of both curriculum learning and adversarial training [14,15], a novel framework named CGAL is constructed. This framework dramatically enhances the model’s performance by capitalizing on curriculum learning to expedite convergence toward an optimal local solution, while incorporating adversarial training to significantly reinforce the Pillar-RBFN’s adversarial robustness. The experimental results show that it achieves an improvement of 0.8∼2.5 percentage points in mAP compared to the normal training on PointPillars. In addition, to alleviate the class imbalance affecting the original KITTI 3D object detection dataset [11], our method introduces a single-frame ground truth (GT) augmentation technique named SFGTS, which creates a complementary dataset by sampling copious object meshes into the original KITTI dataset, resulting in the generation of its adversarial version, Adv-KITTI. This dataset can also be utilized to improve the other peer detectors, which can achieve an enhancement of at least 15 percentage points in mAP. Finally, to further mitigate the class imbalance issue, a self-adaption version of focal loss is formulated, which can direct the model’s attention to challenging objects during the training process. All the contributions are summarized as follows:

- A curriculum-guided adversarial learning framework named CGAL is proposed, which progressively raises the difficulty of adversarial examples during training, enabling the model to evolve from its state to maturity.
- A 3D object detector named Pillar-RBFN is developed based on PointPillars, which intrinsically possesses adversarial robustness without undergoing adversarial training, due to the incorporation of a designed nonlinear enhancement block.
- To train the Pillar-RBFN adversarially, our method further proposes a novel method, P-FGSM, to generate adversarial perturbations, which can be inserted into the original point clouds to produce diverse adversarial examples.
- In order to mitigate the class imbalance issue within the KITTI dataset, this paper further designs an adversarial dataset based on KITTI, which is referred to as Adv-KITTI, by implementing a single-frame ground truth augmentation technique named SFGTS, which creates a complementary dataset of copious object meshes.
- A novel variant of focal loss is formulated, which allows the developed detector to distinguish between challenging and simple objects, substantially intensifying the attentiveness to strenuous ones through the adaptive modulation of hyperparameters.

The remainder of this article is organized as follows. Section 2 reviews the relevant research related to this work. Section 3 provides the background information, including the preliminary tasks and the motivations behind this research. The proposed method is elaborated on in Section 4, while the experimental details are presented in Section 5. Finally, the entire research work is discussed in Section 6 and our conclusions are presented in Section 7, respectively.

## 2. Related Works

This section introduces the tasks relevant to our study. It begins with an elaboration on various LiDAR-based 3D object detectors and adversarial attack methods in the domain of 2D image vision. Following this, several tasks exploring adversarial attacks on LiDAR object detection are discussed.

### 2.1. LiDAR-Based 3D Object Detection

In contrast to 2D images, the processing of 3D point clouds presents a more intricate level of complexity [1]. The fractionalized handling of point clouds has led to the development of distinct branches of 3D detectors, principally classified into point-based, grid-based, and hybrid approaches that synergistically combine the former two paradigms.

Point-based methods [6,19,20], directly cater to the point cloud by initially downsampling the data and subsequently extracting abstract point features by specialized backbone networks, such as the PointNet series [21,22]. Finally, 3D bounding boxes are predicted based on the downsampled points throughout their corresponding features. For the majority of such detectors, the principal bottleneck regarding inference time stems from the point cloud downsampling process (e.g., the furthest point sampling, FPS [22]), which incurs substantial memory access overhead [6].

The grid-based methods inaugurate the process by rasterizing the point cloud into a discrete grid representation, encompassing voxels [23,24,25] and columns [5]; after this, a conventional 2D convolutional neural network comes in handy for feature extraction, culminating in the retrieval of 3D objects from a bird’s-eye view (BEV) grid. However, the transition of continuous point coordinates into a discrete grid inevitably leads to the loss of certain 3D information, which is sensitively influenced by the size of the grid cells. Smaller grids facilitate higher resolution, but this reduction in cell size precipitates a quadratic increase in memory consumption [26].

The hybrid methods tactfully harness the fortes of both point and voxel representations—specifically, the rich spatial information offered by point methods and the efficient memory access provided by voxel methods. Differentiating their detection processes, these approaches can be categorized as one-stage and two-stage models. The two-stage models [7,27,28,29] begin with generating a collection of 3D candidate boxes (i.e., 3D proposals) that potentially encapsulate the positive objects and follow a refinement process. In contrast, the one-stage methods [26,30,31] perform detection directly on the entire point cloud or voxel representation in a straight line, discarding the explicit generation of candidate boxes, which sacrifices detection accuracy in favor of accelerated inference.

### 2.2. Adversarial Attack on 2D Image Vision

In 2013, Szegedy et al. [32] pioneeringly identified the susceptibility of neural networks to adversarial perturbations, revealing that the application of minor disturbances to image pixels can substantially alter the model’s classification results. They designated these subtly altered inputs, which remain indistinguishable to the human eye yet provoke misclassification by the model, as adversarial examples. Afterward, a series of transformative methods have emerged, mainly built upon this milestone [33].

FGSM [9] and FGM [34] engineered adversarial examples utilizing a single gradient step, which perturbs the model’s input prior to executing backpropagation. Subsequently, Moosavi-Dezfooli et al. [35] proposed DeepFool, which calculates the minimal necessary perturbation and applies it to construct adversarial examples, employing the $L_{2}$ norm to constrain the magnitude of perturbations. Concurrently, Papernot et al. [36] introduced the Jacobian saliency map attack (JSMA), which fabricates adversarial examples by computing the derivatives during the neural network’s forward propagation. To rectify the linearity assumption existing in both FGSM [9] and FGM [34], Madry et al. [18] presented the projected gradient descent (PGD) method to address the underlying maximization challenge. C&W [37] developed three innovative attack algorithms targeting the $L_{0}$, $L_{2}$, and $L_{\infty }$ distance metrics, demonstrating that defensive distillation [38] fails to enhance the adversarial robustness of neural networks.

The attack methods mentioned above rely on the gradient information of the victim model, which implicitly provides the attacker with a comprehensive understanding of the model’s structure; thus, these methods are regarded as white-box attacks. Contrarily, black-box attacks operate under the assumption that the victim model remains impervious to scrutiny. Chen et al. [39] employed a zeroth-order optimization strategy that directly estimates the first-order and second-order gradient information of the victim model. This approach enables the simulation of attacks in real-world scenarios. Papernot et al. [40] proposed substitute training (ST), which builds upon the principles of transfer attacks to generate adversarial examples, which can be tailored to the victim model, enhancing the attack success rate. Brendel et al. [41] introduced the boundary attack (BA), which employs a closed-loop process of continuous sampling, perturbation, and querying to identify an adversarial example that closely resembles the original sample, which prevents the examples’ shape from severe distortion. Unlike most peers, He et al. [42] designed a novel algorithm that leverages modified attention heatmaps to generate adversarial examples, demonstrating its effective transferability to feasibly attack other victim models. Li et al. [43] presented a novel framework named the content-based unrestricted adversarial attack (CUAA), which utilizes a stable diffusion model to generate transferable unrestricted adversarial examples with various adversarial contents. Due to the strength of the diffusion model, it can generate vibrant and colorful adversarial images.

### 2.3. LiDAR Detectors with Adversarial Attack

In the LiDAR-based 3D object detection domain, research concerning adversarial attacks remains relatively scarce and a meticulously curated summary of our findings is presented as follows: Lehner et al. [44] proposed 3D-VField, a data augmentation method that enhances generalization by plausibly deforming point clouds using adversarially learned vector fields. Tu et al. [45] and Abdelfattah et al. [10] manufactured adversarial examples by strategically positioning adversarial objects atop target vehicles. The efficacy of these attacks is primarily attributed to the scarcity of pertinent training data—a limitation that can be alleviated through suitable data augmentation techniques. Employing a differentiable technique, Tu et al. [46] formulated an adversarial textured mesh designed to compromise various multi-sensor 3D detectors by seamlessly rendering the mesh into both LiDAR and image inputs. Cao et al. [8] and Sun et al. [47] implemented a hardware-based technique to generate synthetic points, which are then integrated into the point cloud, thus misleading the perception module into detecting illusory obstacles. Cai et al. [48] proposed a deep autoencoder-based anomaly detection method, which has a strong ability to detect elaborate adversarial examples in an unsupervised way and introduce an augmented memory module with typical normal patterns recorded to improve the performance of the autoencoder. Zhu et al. [49] presented AE-Morpher (AEM), which minimizes differences between the LiDAR-captured and original adversarial point clouds to improve the adversarial robustness of adversarial examples, and it reconstructs the adversarial examples by utilizing surfaces with regular shapes to fit the discrete laser beams.

## 3. Background

In this section, an overview of two preliminaries is briefly introduced, namely, PointPillars [5] and FGSM [9], according to our research context. Following that, the motivations behind this article are elucidated.

### 3.1. Preliminaries I: PointPillars

**Figure 2:**
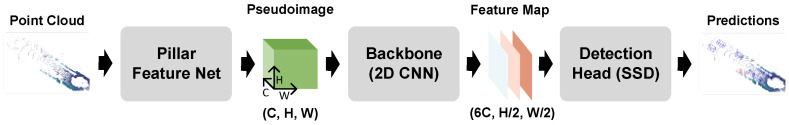
An overview of PointPillars [5], where each component performs its designated function. The first module’s pillar feature net (PFN) compresses the 3D point cloud into a 2D pseudo-image tensor with the shape (C, H, W). The second module’s backbone extracts features from the pseudo-image, which obtains a high-level feature map tensor with the shape (6C, H/2, W/2). The third module’s detection head (DH) utilizes the feature map to predict the 3D bounding boxes of point cloud objects. Parameter C signifies the number of channels, while H and W correspond to the height and width of the pseudo-image, respectively.

PointPillars [5] is an end-to-end LiDAR-based 3D object DeepNet model (i.e., 3D object detector) that forages a point cloud to predict 3D bounding boxes of three categories (i.e., car, cyclist, and pedestrian). As depicted in Figure 2, it consists of three modules: (1) a 3D module pillar feature net (PFN) that converts one frame of the point cloud into a pseudo-image-like 2D feature representation tensor with the shape (C, H, W); (2) a 2D convolutional backbone network that processes the pseudo-image-like representation generated from the first module’s PFN into high-level feature map tensors with the shape (6C, H/2, W/2); and (3) a detection head (DH) that forages the feature maps synthesized from the former module’s backbone to predict the location of the objects’ 3D bounding boxes and class probabilities. Parameter C signifies the number of channels, while H and W correspond to the height and width of the pseudo-image, respectively. In order to utilize the conventional 2D convolutional network to extract features, while considering the inherent three-dimensional nature of point clouds, the researchers behind PointPillars [5] employed the first module PFN to compress the point cloud into a tensor representation akin to the representation of a 2D image, which can be considered a pseudo-image. To avoid redundancy, the remaining parts of this section provide a succinct overview of the loss function required for our research. Further details can be found in [5].

Similar to [24], the total loss function used in PointPillars [5] consists of three components: $L_{reg}$, $L_{cls}$, and $L_{dir}$. The localization loss, $L_{reg}$, regresses the 3D boxes, utilizing the SmoothL1 form [50]:

(1) $$L_{reg}=\sum_{b}SmoothL_{1}(\Delta b)=\left\{\begin{array}{c} 0.5\;{\left(\Delta b\right)}^{2},\;\;\mathrm{if}\left|\Delta b\right|<1\\ \left|\Delta b\right|-0.5,\;\mathrm{otherwise} \end{array}\right.$$

The scalar b belongs to the 3D box parameter set {*x*, *y*, *z*, *w*, *l*, *h*, θ}, where the ordered pair (*x*, *y*, *z*) denotes the geometric center of the box, while θ and the pair (*w*, *l*, *h*) represent the orientation and scale, respectively. Δb indicates the localization residual:

(2) $$\begin{array}{cc} \; & \Delta x=\frac{x_{gt}-x_{a}}{d_{a}},\;\;\Delta y=\frac{y_{gt}-y_{a}}{d_{a}},\;\;\Delta z=\frac{z_{gt}-z_{a}}{h_{a}},\\ \; & \Delta w=log\frac{w_{gt}}{w_{a}},\;\;\;\;\;\;\Delta h=log\frac{h_{gt}}{h_{a}},\;\;\;\;\;\;\Delta l=log\frac{l_{gt}}{l_{a}},\\ \; & \Delta \theta =sin(\theta_{gt}-\theta_{a}) \end{array}$$

where the variables with the corner labels gt and *a* denote the values of the ground truth 3D boxes and anchors, respectively, and $d_{a}=\sqrt{{\left(w_{a}\right)}^{2}+{\left(l_{a}\right)}^{2}}$. For object classification loss, $L_{cls}$, which employs the focal loss, we have the following [51]:

(3) $$L_{cls}=-\alpha_{a}{\left(1-p_{a}\right)}^{\gamma }log\left(p_{a}\right)$$

where $p_{a}$ denotes the classification probability of an anchor, assigning α=0.25 and γ=2 originally [5]. In particular, considering that Δθ=0 in Equation (3) when the object orients at either $0^{\circ }$ or $180^{\circ }$ in reality, $L_{dir}$ compensates for this deficiency by utilizing the softmax classification loss. Ultimately, the total loss attains the following formula:

(4) $$L_{tot}=\frac{1}{N_{pos}}\left(\beta_{reg}L_{loc}+\beta_{cls}L_{cls}+\beta_{dir}L_{dir}\right)$$

where $N_{pos}$ denotes the number of positive anchors while $\beta_{reg}=2$, $\beta_{cls}=1$, and $\beta_{dir}=0.2$. As illustrated in Table 1, the implementation of voxelization for point clouds, coupled with the utilization of a lightweight feature extraction network, enables PointPillars [5] to occupy comparatively less memory overhead than other similar approaches, rendering it exceptionally favored in practical industrial applications.

### 3.2. Preliminaries II: FGSM

The fast gradient sign method (FGSM) [9] serves as a commonly explored technique for generating adversarial examples, which evaluates model adversarial robustness by reducing predictive accuracy. The fundamental principle of FGSM [9] involves consulting the model’s gradient information with respect to the input to create minimal yet impactful perturbations, thus misleading the model into producing incorrect predictions on adversarial samples, and it produces adversarial perturbations δ through the following formula:

(5) $$\delta =\epsilon \cdot sign(\nabla_{x}J\left(\theta ,x,y\right))$$

where *x* denotes the original input, y indicates the true label, θ represents the model parameters, and $\nabla_{x}J\left(\theta ,x,y\right)$ signifies the gradient of the loss function J with respect to *x*.

As a single-step attack, one notable strength of the FGSM [9] lies in its operational efficiency, and it owns the capability to generate diverse examples. For instance, by increasing the intensity of the perturbations (i.e., adjusting the value of ϵ), it becomes possible to deliver more pronounced adversarial instances. Furthermore, benefiting from the foundational principles, FGSM [9] can be synergistically combined with other methodologies, such as iterative approaches, to yield more intricate adversarial examples.

### 3.3. Motivation

The rapid advancement of deep learning architectures has profoundly improved the performance of 3D object detection models. However, recent studies [8] reveal that these models exhibit notable vulnerabilities to adversarial attacks. The majority of LiDAR-based 3D object detectors, such as PointPillars [5], tend to overlook this critical issue, which may potentially lead to severe safety incidents in autonomous vehicles due to the widespread application of these technologies in reality.

FGSM [9] provides an elegant and efficient framework for crafting adversarial examples. Consequently, our research endeavors to leverage this capability to enhance the adversarial robustness of PointPillars [5]. It is believed that integrating FGSM [9] with 3D object detection not only facilitates a deeper understanding of the inherent vulnerabilities within these models but also paves the way for the development of enhanced training strategies geared toward fortifying model resilience, contributing to the creation of safer and more dependable 3D object detection systems in realistic applications, such as the pitfalls mentioned in Section 1.

## 4. Methodology

In this section, a detailed exposition of each component within the proposed framework is provided, aiming to augment the clarity of the technical contributions delineated by the research.

### 4.1. Hierarchical Sample Partition

**Figure 3:**
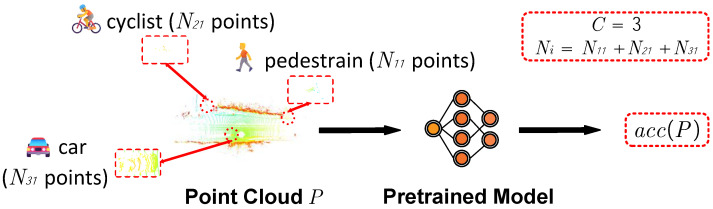
An illustration of the hierarchical sample partition, where C represents the number of categories in the current point cloud *P*, and $N_{i}$ denotes the number of points in the i-th category. The variables *C* and *N* are calculated based on the ground truth objects within *P*, and acc is attained from the pre-trained model, which utilizes the mean average precision (mAP) item. The pre-trained model entrusts a cloned PointPillars [5] trained on the nuScenes dataset [52].

The configuration of neural networks succinctly manifests the operational principles in the human brain. Human learning typically follows a progression from simple to complex concepts. Similarly, the curriculum-learning principles indicate that adhering to a process that resembles human learning can enhance the abilities of DeepNet to adapt to previously unseen data [15]. As a consequence, our study intends to utilize it for reference, which arranges the training split of the KITTI dataset [11] according to a specific criterion, one of which is universally applicable as follows:

(6) $$s=\alpha \cdot C+\beta \cdot \sum_{i=1}^{C}N_{i}\;s.t.\;\alpha ,\beta \in \left(0,1\right)$$

where C represents the number of categories in the current frame, $N_{i}$ denotes the number of points in the i-th category, and α and β denote the balancing factors of the corresponding item. Nevertheless, our research asserts that this solution is fundamentally subjective, as genuine insights ought to be derived from those with firsthand experiences. Consequently, our criteria are further combined with an accuracy item, which is defined as follows:

(7) $$\bar{s}=s+\gamma \cdot acc\left(P\right)\;s.t.\;\gamma \in \left(0,1\right)$$

where acc(P) denotes the accuracy performance of the current frame *P* on the pre-trained model, and γ is analogous to α and β. This study utilizes mean average precision (mAP) as the accuracy item and denotes state $\bar{s}$ as the detection difficulty of the current frame, where a higher value indicates a simpler detection level. Figure 3 illustrates the processing of the hierarchical sample partition (HSP). The pre-trained model employs a cloned PointPillars network [5] trained on the nuScenes dataset [52] and our task focuses exclusively on the detection of three categories: car, cyclist, and pedestrian.

### 4.2. Generation of Adversarial Examples

FGSM [9] has emerged as a pivotal tool in the realm of adversarial attack research, pervasively employed to assess the adversarial robustness of machine learning models and evaluate the generation capabilities of adversarial examples; its popularity is attributed to its high efficiency, straightforward implementation, and robust effectiveness, requiring only one forward and backward propagation. This approach possesses rapid computation speeds suitable for real-time applications. Consequently, in order to align with the current task, our research proposes a variant, P-FGSM, based on this method, which utilizes LiDAR point clouds to generate adversarial perturbations:

(8) $$\bar{\delta }=\epsilon \cdot sign\left(\nabla_{P}J\left(\theta ,P,y\right)\right)⊙D$$

where $P={\left\{p_{i}\right\}}_{i=1}^{\left|P\right|}$ represents the current input point cloud, while D denotes the positional weight matrix that reflects the significance and spatial relationships of each point. To improve the convergence of the training process, our method selects to dynamically adjust the perturbation parameter ϵ based on the model’s detection feedback, which is scaled according to the $L_{2}$ norm of the current gradient $\nabla_{P}J\left(\theta ,P,y\right)$:

(9) $$\epsilon_{t}=\epsilon_{t-1}-sign\left(\nabla_{P}J\right)\cdot {∥\nabla_{P}J∥}_{2}$$

where *t* denotes the current iteration step and $\nabla_{P}J$ is the abbreviation for $\nabla_{P}J\left(\theta ,P,y\right)$. After analyzing Equation (9), it can be seen that when the model loss declines, the gradient is negative, which allows for an increase in the perturbation magnitude, which enhances the arduousness of detection, and the reverse applies conversely. Intuitively, objects in closer proximity to the vehicle necessitate more precise capture due to their paramount importance to driving safety. Our research assigns greater weight, $d_{n}$, to points that are closer than the more remote $d_{r}$, indicating more pronounced perturbations, while foreground points receive even higher weights, $d_{f}$, than the background weight, $d_{b}$; the specifics are as follows:

(10) $$D={\left\{d|d_{n}>d_{r}\cap d_{f}>d_{b}\right\}}_{\left|P|\times |F\right|}$$

where |F| denotes the feature dimension associated with one point p. Given that the 3D bounding box separates objects from the background, our study employs a segmentation model, PointNet [21], to function as an attacker model; $J_{f}$ denotes the weighted sum of the classification cross-entropy loss, the 3D bounding box regression loss, and the semantic loss, $L_{sem1}$, from the attacker for points belonging to the object set $P_{f}$ (i.e., foreground points). For points associated with the background $P_{b}$ lacking annotations, our research commissioned a model Cylinder3D [53] as the attacker and utilized its semantic cross-entropy loss as $L_{sem2}$:

(11) $$J=\left\{\begin{array}{cc} J_{f}=\zeta_{1}\left(\bar{L_{cls}}+L_{reg}\right)+\zeta_{2}L_{sem1}, & \mathrm{if}\;\;\;p_{i}\in P_{f}\\ J_{b}=L_{sem2}, & \mathrm{if}\;\;\;p_{i}\in P_{b} \end{array}\right.$$

where $\zeta_{1}$ and $\zeta_{2}$ represent balancing factors, while $\bar{L_{cls}}$ and $L_{reg}$ are defined in Section 3.1 and Section 4.5, respectively. It is imperative to note that *J* obtains the term $\bar{L_{cls}}+L_{reg}$ through the model’s intrinsic training, while it remains unable to determine the term $L_{sem1}$. Thus, this study introduces balancing factors $\zeta_{1}$ and $\zeta_{2}$ to reconcile the information from both white-box and black-box perspectives. After that, the foreground gradient $\nabla_{P}J_{f}$ and background gradient $\nabla_{P}J_{b}$ can be obtained, and the adversarial perturbations $\bar{\delta }$ can be divided into two parts, correspondingly, i.e., the perturbations of foreground points $\bar{\delta_{f}}$ and background points $\bar{\delta_{b}}$:

(12) $$\nabla_{P}J=\left\{\begin{array}{cc} \nabla_{P}J_{f}=\nabla_{P}J\left(\zeta_{1}\left(\bar{L_{cls}}+L_{reg}\right)+\zeta_{2}L_{sem1}\right), & \mathrm{if}\;\;\;p_{i}\in P_{f}\\ \nabla_{P}J_{b}=\nabla_{P}J\left(L_{sem2}\right), & \mathrm{if}\;\;\;p_{i}\in P_{b} \end{array}\right.$$

(13) $$\bar{\delta }=\left\{\begin{array}{cc} \bar{\delta_{f}}=\epsilon \cdot sign\left(\nabla_{P}J_{f}⊙D\right), & \mathrm{if}\;\;\;p_{i}\in P_{f}\\ \bar{\delta_{b}}=\epsilon \cdot sign\left(\nabla_{P}J_{b}⊙D\right), & \mathrm{if}\;\;\;p_{i}\in P_{b} \end{array}\right.$$

Finally, the adversarial version $\bar{P}$ of the current frame, *P*, is obtained by further adding the generated adversarial perturbations into the original point cloud, *P*:

(14) $$\begin{array}{cc} \bar{P_{f}} & =P_{f}+\bar{\delta_{f}} \end{array}$$

(15) $$\begin{array}{cc} \bar{P_{b}} & =P_{b}+\bar{\delta_{b}} \end{array}$$

(16) $$\begin{array}{cc} \bar{P} & =\bar{P_{f}}+\bar{P_{b}} \end{array}$$

**Figure 4:**
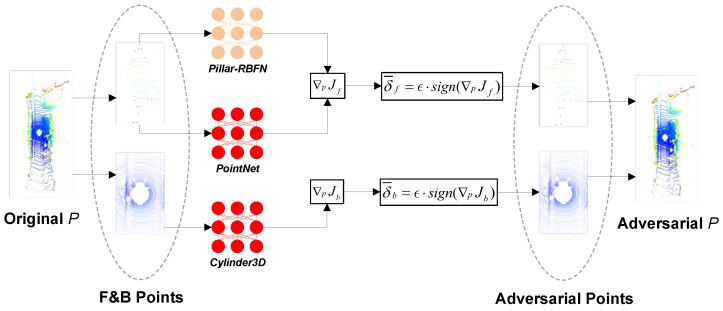
The generation flow of adversarial examples [24] utilizing P-FGSM on the current frame, *P*. The left side, indicated by the gray dashed lines, represents the foreground point (a pedestrian in the top line) alongside the background points (bottom line). The red models, PointNet and Cylinder, are entrusted as the attackers, while the beige Pillar-RBFN serves as the victim model. The variables $\nabla_{P}J_{f}$ and $\nabla_{P}J_{b}$ denote the foreground and background gradients, respectively, as defined in Equation (12). Furthermore, $\bar{\delta_{f}}$ and $\bar{\delta_{b}}$ represent the adversarial perturbations, as specified in Equation (13).

**Figure 5:**
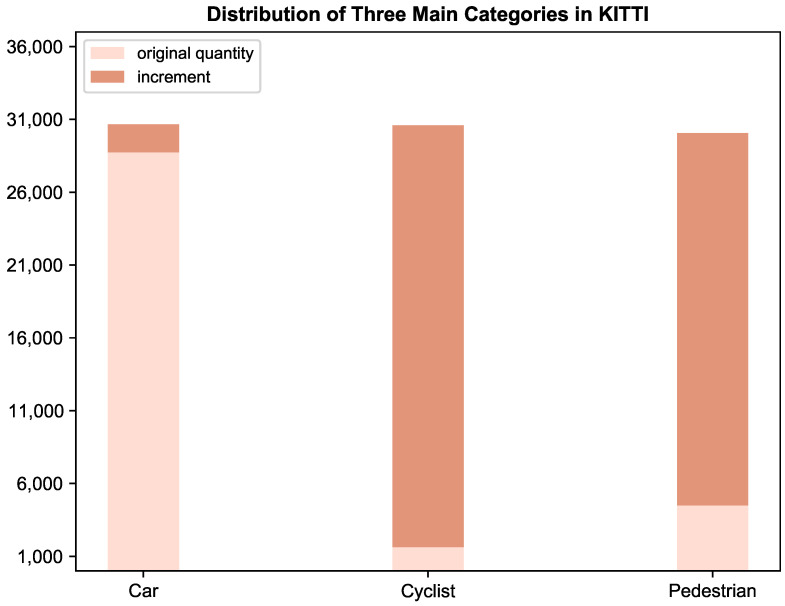
The distribution of three main categories in the KITTI 3D object detection dataset [11] where the bars with lighter colors represent the original quantities, the bars with deeper colors indicate the increments, and the sum bars indicate the quantities of the new adversarial dataset, Adv-KITTI. After implementing the single-frame ground truth sample (SFGTS) mentioned in Algorithm 1 for frame-by-frame sampling on the original KITTI dataset, a substantial increase is achieved in the previously scarce instances of cyclists and pedestrians, bringing their numbers on par with the car class. Since the original KITTI dataset primarily consists of cars, the augmentation effect is comparatively weaker for this category.

**Figure 6:**
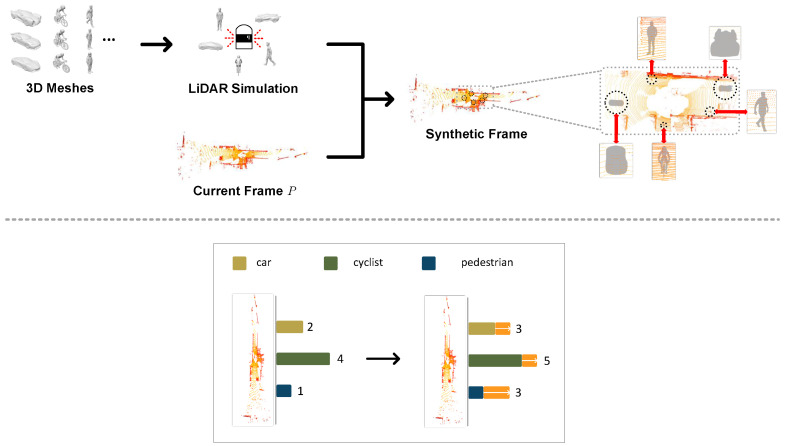
The processing flow of the single-frame ground truth sample (SFGTS) [24] utilizing Algorithm 1 on the current frame, *P*. During the sampling process, the algorithm randomly selects a specified number of targets from the mesh dataset based on the quantity of the three object types present in the current point cloud scene, *P*; these objects are subsequently converted into point clouds through LiDAR simulation and integrated into *P*. The bottom diagram indicates that for the number of objects in all three categories (i.e., car, cyclist, and pedestrian), point cloud *P* universally increased after augmenting; it can be seen that the SFGTS algorithm samples 1 car, 1 cyclist, and 2 pedestrians into it. The 3D meshes were collected in advance and the LiDAR simulated 64 lines corresponding to the KITTI dataset.

The whole generation process can also be seen in Figure 4. Moreover, in light of the class imbalance within the origin KITTI dataset [11], particularly with the dominance of the car category as depicted in Figure 5, our research prepared an additional 3D object mesh dataset, where it randomly sampled the scarce objects into the current frame *P* before the generation process, as illustrated in Figure 6. During the sampling process, a specified number of objects were randomly selected from the 3D mesh dataset based on the quantities of the three object categories (i.e., car, cyclist, and pedestrian) present in the current point cloud scene. These objects were then converted into point clouds and subsequently integrated into *P*. It can be seen that the algorithm samples one car, one cyclist, and two pedestrians to synthesize the final point cloud. Figure 5 illustrates the distribution of three main categories of the whole KITTI dataset [11] before and after the SFGTS process (Adv-KITTI).
**Algorithm 1** Single-frame ground truth sample**Initialization:  **minimum quantity $\theta_{min}$ ∈ {$\theta_{min1}$, $\theta_{min2}$, $\theta_{min3}$}, maximum quantity $\theta_{max}$ ∈ {$\theta_{max1}$, $\theta_{max2}$, $\theta_{max3}$};**Inputs:  **Point Cloud *P*, Mesh Dataset M; 1:Scan The current frame *P* to obtain the quantity of the object car $q_{car}$, cyclists $q_{cyc}$, and pedestrians $q_{ped}$; 2:$q\to \{q_{car},q_{cyc},q_{ped}\}$, $q^{\prime }\to \left\{q_{1},q_{2},q_{3}\right\}$; 3:**while** 1 ≤ *i* ≤ 3 **do** 4:   **if** $0\leq q\left[i\right]<\theta_{min}\left[i\right]$ **then** 5:     $q^{\prime }\left[i\right]\to \theta_{min}\left[i\right]$ 6:   **else** 7:     **if** $\theta_{min}\left[i\right]\leq q\left[i\right]<\theta_{max}\left[i\right]$ **then** 8:        $q^{\prime }\left[i\right]\to Rand\left(q\left[i\right],\;\;\theta_{max}\left[i\right]+1\right)$; 9:     **end if**10:  **end if**  $m=SampMesh(q^{\prime }\left[i\right]-q\left[i\right])$;  $P^{\prime }=LidarSimu\left(P,\;\;m\right)$;11:**end while**12:**return** $P^{\prime }$

Following the implementation of the SFGTS, it can be observed that the original dataset obtains a substantial increase in the previously scarce instances of cyclists and pedestrians, bringing their counts on par with the car class. Since the original KITTI dataset primarily consists of cars, the augmentation effect is comparatively weaker for this category.

### 4.3. Nonlinear Enhancement Block

**Figure 7:**
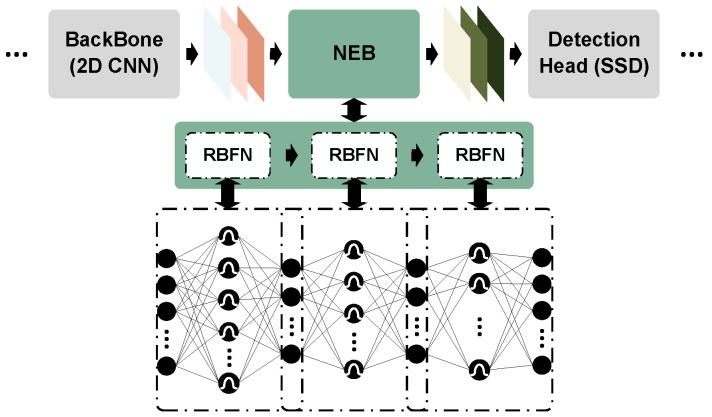
An illustration of the nonlinear enhancement block (NEB), which constructs radial basis function networks (RBFNs) [54] with varying numbers of hidden neurons utilizing Gaussian kernels. The first module receives a tensor with the shape (6C, H/2, W/2) from the backbone and reshapes it with the shape (6C, HW/4), with the neuron counts in its three layers set at HW/4, HW/2 and HW/8, respectively. The second module embraces the former tensor with the shape (6C, HW/4), comprising three layers, with neuron counts of HW/8, HW/4 and HW/8, respectively. The third module devours the output from the previous layer, maintaining the output tensor’s shape at (6C, HW/4); the output is then reshaped back to shape (6C, H/2, W/2) for input into the detection head module, with neuron counts in its three layers specified as HW/8, HW/6, and HW/4, respectively.

**Figure 8:**
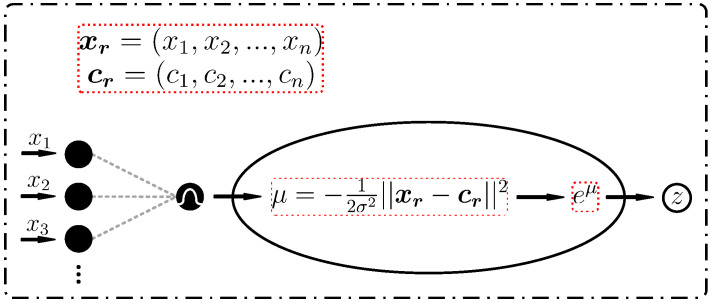
An illustration of one Gaussian kernel hidden neuron, where $x_{r}$ represents the input vector for the $r_{th}$ hidden layer neuron, $c_{r}$ denotes the center vector determined by the k-means clustering, and *z* indicates the output. σ is the width parameter of the Gaussian kernel, which governs the response scope. The RBFN equipped with a Gaussian kernel function can achieve nonlinear mapping and has a very strong function approximation capability, which is committed to strengthening the nonlinearity of PointPillars [5] in our method.

Deeper neural networks exhibit adversarial robustness to adversarial examples. However, as highlighted by [9], nonlinearity is the primary factor underlying their susceptibility to adversarial attacks, and enhancing the model’s nonlinearity substantially strengthens the adversarial resilience. The radial basis function network (RBFN) [54] can achieve complex nonlinear mappings through its multilayer architecture when integrated as a module within other models. Furthermore, the local characteristics of RBFN [54] render it relatively resilient to input noise, enabling it to withstand adversarial attacks. Our research leverages PointPillars [5] as the foundational model and incorporates three RBFN [54] modules between the 2D backbone and the detection head to augment depth and nonlinearity. Specifically, the Gaussian kernel is employed for the transformation of feature dimensions in these three modules:

(17) $$\phi \left(x_{r}\right)=e^{-\frac{{\left|x_{r}-c_{r}\right|}^{2}}{2\sigma^{2}}}$$

where $x_{r}$ represents the input vector for the $r_{th}$ hidden layer neuron, $c_{r}$ denotes the center vector, and σ is the width parameter of the Gaussian kernel, governing the response scope. As illustrated in Figure 7, three RBFN [54] modules are sequentially interconnected. The first module receives a feature map tensor of dimensions (6C, H/2, W/2) from the 2D backbone, subsequently reshaping it to (6C, HW/4) for input, with the neuron counts in its three layers set at HW/4, HW/2 and HW/8, respectively. The second module embraces the output from the preceding layer as input, comprising three layers with neuron counts of HW/8, HW/4 and HW/8, respectively. Similarly, the third module devours the output from the previous layer, maintaining the output tensor dimensions at (6C, HW/4); this output is then reshaped back to (6C, H/2, W/2) for input into the detection head module, with neuron counts in three layers specified as HW/8, HW/6, and HW/4, respectively. Figure 8 illustrates one Gaussian kernel hidden neuron, where $x_{r}$ represents the input vector for the $r_{th}$ hidden layer neuron, $c_{r}$ denotes the center vector determined by k-means clustering and *z* indicates the output. σ is the width parameter of the Gaussian kernel, which governs the response scope. The RBFN, equipped with a Gaussian kernel function, can achieve nonlinear mapping and has a very strong function approximation capability, which is designed to strengthen the nonlinearity of PointPillars [5] in our method.

### 4.4. Robustness Training with Curriculum

**Figure 9:**
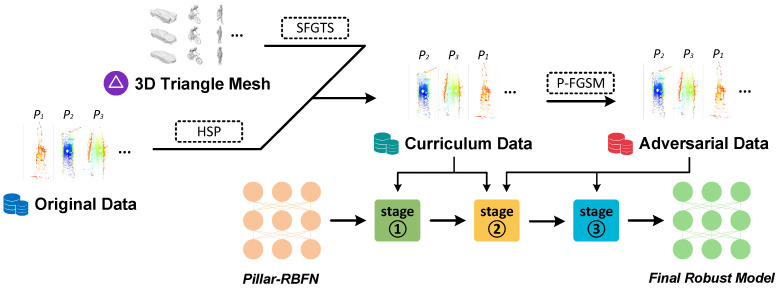
The overview of our curriculum-guided adversarial learning (AGCL) framework, which primarily encapsulates three stages to train Pillar-RBFN. The SFGTS [24] denotes the single-frame ground truth sample obtained by Algorithm 1, HSP refers to the hierarchical sample partition explicated in Section 4.1, and P-FGSM [9] denotes our perturbation method elucidated in Section 4.2. The original data denote the KITTI dataset without augmentation. The 3D triangle mesh is a pre-collected dataset, while the other two datasets (i.e., the curriculum and adversarial data) were derived from or generated within the training split of the KITTI 3D object detection dataset [11]. The curriculum data were engaged for training the Pillar-RBFN during both the first and second stages, while the adversarial data were merely utilized in the latter second and third stages.

**Figure 10:**
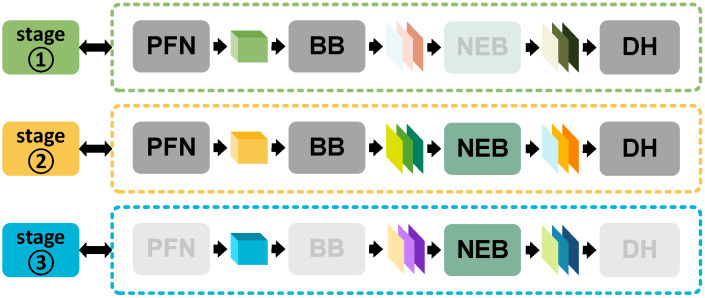
An illustration of the three stages during adversarial training. The modules with 60% transparency remained frozen during the corresponding stage, while the others were updated iteratively, as usual. In the first stage, the PFN, Backbone, and DH modules related to PointPillars [5] were activated, whereas the designed NEB module was frozen. In the second stage, all modules were trained. In the third stage, the three modules of PointPillars were stabilized while the NEB module continued to operate.

In order to construct a robust model (i.e., the designed Pillar-RBFN), this research meticulously formulates a training framework named curriculum-guided adversarial learning (AGCL), as illustrated in Figure 9. It initially utilizes the pre-collected 3D triangle mesh data to further generate point cloud objects with the designed SFGTS and pastes them into the original KITTI dataset. After this, it processes the boosted dataset with the hierarchical sample partition (HSP), and the curriculum dataset is attained, which is essential for the training of Pillar-RBFN during the first and second stages. Finally, the curriculum data are perturbed by our P-FGSM method to generate the adversarial dataset, which is then used to train the Pillar-RBFN during the second and third stages. The framework AGCL encapsulates an engineered training strategy, which consists of the three training stages with their own responsibilities, as depicted in Figure 10, and trains the Pillar-RBFN adversarially with curriculum (CRT). The three stages are illuminated as follows:

- Stage 1: Initial training of PointPillars.

In the first stage, as shown in Figure 9, the three modules related to PointPillars [5] (i.e., the module PFN, Backbone, and DH) are trained with a series of epochs on the scrupulously organized curriculum dataset mentioned in Section 4.1. After reaching a better level of accuracy, the training process accomplishes stage one. During this stage, the weight parameters of the nonlinear enhancement block (NEB), mentioned in Section 4.2, are frozen, as illustrated in the top line of Figure 10.

- Stage 2: Adversarial sample generation and joint training.

In the second stage, the designed NEB modules are activated and begin generating adversarial samples following the methodology mentioned in Section 4.2, as shown in the medium line of Figure 10. Both the adversarial samples and their corresponding original frames are fed into the entire model to iterate the weight parameters, i.e., the NEB and PointPillars are trained jointly. After several epochs, this completes stage two and the model enters the final stage.

- Stage 3: Final training on NEB modules.

In the third stage, the module weights of PointPillars [5] are fairly stabilized, similar to stage one, where the NEB module is frozen, as shown in the bottom line of Figure 10. During this stage, adversarial samples are exclusively utilized to train the NEB modules, as illustrated in Figure 9, for the final several epochs. After this, the entire training process is finished and the baked Pillar-RBFN model can be obtained.

The whole training process surrounding Pillar-RBFN can be queried in Algorithm 2, where the relative settings of the parameters are introduced in Section 5. Curriculum learning effectively mitigates severe fluctuations in loss, accelerating the model’s convergence speed. By structuring the original training split into a coherent curriculum, the first stage of training PointPillars [5] swiftly and smoothly converges on a superior local optimum. In the second stage, the NEB modules achieve convergence, imparting significant adversarial robustness to both itself and the PointPillars [5] network. In the final stage, it strategically freezes the weights of PointPillars [5], as our method assumes the critical role of learning additional adversarial patterns to counteract potential attacks forcefully.
**Algorithm 2** Robust adversarial training.**Initialization:  **exponential factor $\gamma_{min}$, sensitivity factor *k*;**Inputs:  **training split $D_{tra}$, validation split $D_{val}$, epoch *e*;1:$\gamma_{min}\to 1.5$, k→40;2:**for** 0≤e≤100 **do**3:   Substitute the loss function $L_{cls}$ in Equation (3) for Equation (18) on $D_{tra}$;4:   Update γ and *k* by Equation (21) and Equation (22), respectively;5:   **while** 0≤e<25 **do**6:     Train with $D_{tra}$;7:     Fix the NEB module;8:   **end while**9:   **while** 25≤e<75 **do**10:     Release the NEB module;11:     Obtain the adversarial split $D_{tra}^{\prime }$ by Equation (14);12:     Train with $D_{tra}^{\prime }$ and $D_{tra}$;13:   **end while**14:   **while** 75≤e≤100 **do**15:     Fix the other three modules except for the NEB module;16:     Train with $D_{tra}^{\prime }$;17:   **end while**18:**end for**19:Evaluate with $D_{val}$20:**return** Final robust model

### 4.5. Adaptive Class-Balanced Loss

In the community of LiDAR-based 3D object detection, a considerable imbalance exists between the foreground and background points. Despite the SFGTS mentioned above, which can address inter-class quantity disparities, it fails to mitigate the intra-class imbalance that mainly refers to the density distinction between challenging and simple objects. The researchers behind PointPillars [5] employed focal loss to tackle this issue. However, it typically relies on fixed hyperparameters, which may hinder the model from learning sufficiently from particularly challenging objects. Consequently, this study devises slight modifications based on it by introducing a dynamic sample difficulty adaptation mechanism:

(18) $$\bar{L_{cls}}=-\alpha \left(p_{c}\right)\cdot {\left(1-p_{c}\right)}^{\gamma (p_{c})}\cdot \log\left(p_{c}\right)$$

The manual adjustment of hyperparameters regularly exhibits blindness, and the process of identifying the optimal combination appears exceptionally time-intensive. One straightforward approach involves treating α and γ as variables and updating them concurrently with the network weight parameters:

(19) $$\alpha_{j+1}\leftarrow \alpha_{j}-\eta \cdot \frac{\partial \bar{L_{cls}}}{\partial \alpha }$$

(20) $$\gamma_{j+1}\leftarrow \gamma_{j}-\eta \cdot \frac{\partial \bar{L_{cls}}}{\partial \gamma }$$

**Figure 11:**
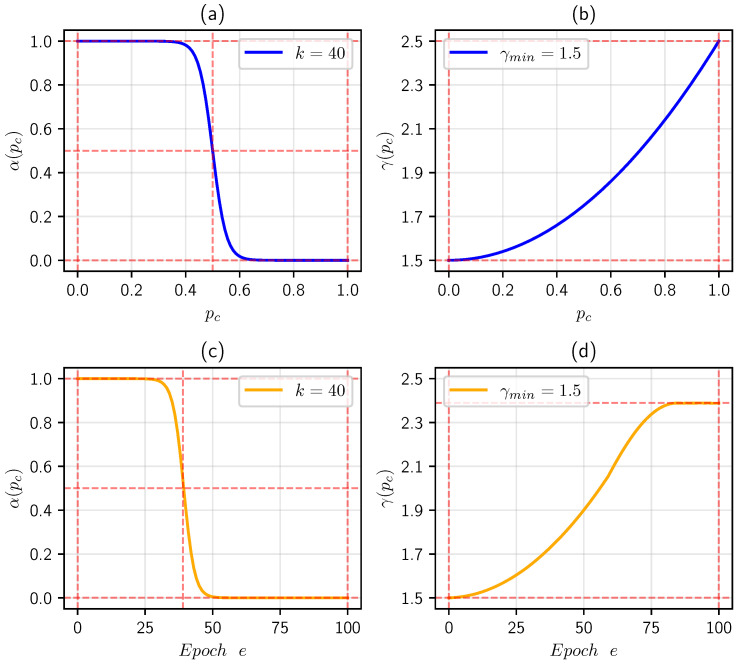
The function charts of the adaptive parameters α and γ in diagrams (**a**,**b**), along with their distribution of values during the training process (ϵ=5) in diagrams (**c**,**d**). The α and γ are defined in Equation (21) and Equation (22), respectively; $p_{c}$ refers to the confidence scores of the object’s corresponding category, while *k* and γ are hyperparameters that are equal to 40 and 1.5, respectively. It can be observed from diagrams (**c**,**d**) that as training progresses, parameters α and γ essentially satisfy the theoretical function relationship in (**a**,**b**).

However, this approach evidently presupposes that α and γ are collectively influenced by both challenging and simple samples. In actuality, for challenging samples, α ought to be larger, whereas γ matures comparatively smaller. Conversely, for easily distinguishable objects, the inverse relationship applies. As a consequence, this research proposes establishing parameters α and γ to dynamically adapt to the confidence scores $p_{c}$ of their respective categories:

(21) $$\alpha \left(p_{c}\right)=\frac{1}{1+\exp[k\cdot \left(p_{c}-0.5\right)]}$$

(22) $$\gamma \left(p_{c}\right)=\gamma_{min}+{\left(p_{c}\right)}^{2}$$

where k serves to regulate the sensitivity of α, while $\gamma_{min}$ represents the minimum value of γ. Here, our study sets k=40 and $\gamma_{min}=1.5$, as analyzed in Section 5.3.2. It is noteworthy that as $p_{c}$ escalates, the parameter α decreases and γ behaves conversely. Figure 11, diagram (a), illustrates the functional relationship between α and $p_{c}$. It is evident that trickier objects ($p_{c}\in \left(0.0,\;0.4\right)$) receive higher weights (approximately equal to 1), and the weights of objects with moderate difficulty ($p_{c}\in \left(0.4,\;0.6\right)$) exhibit a gradual decline with respect to $p_{c}$ while those of easier identifiable objects ($p_{c}\in \left(0.6,\;1.0\right)$) converge toward zero. Figure 11, diagram (b), depicts the functional relationship between γ and $p_{c}$. It is conspicuous that γ increases with $p_{c}$ and objects with higher $p_{c}$ are assigned greater values of γ, theoretically, resulting in a reduced contribution to the overall loss. For instances with low confidence, the loss is magnified relatively, which directs the model’s attention toward knottier ones. By establishing a direct correlation between α and γ and the confidence levels, it enables simple computation before each forward propagation and facilitates the model to adaptively refine its focus when processing diverse samples.

## 5. Experiment

This section is dedicated to detailing the structure of the experiments along with the results, which are presented to emphasize the contributions of our research.

### 5.1. Dataset and Preprocessing

The official guidelines delineate the KITTI 3D object detection dataset [11] into two distinct subsets: a training set comprising 7481 frames of point clouds and their corresponding 2D images, and a test set containing 7561 frames. This dataset encompasses three main categories: pedestrian, car, and cyclist. Within each category, three distinct detection levels are established, i.e., easy, moderate, and difficult levels, which are determined by various factors, including object size, degree of occlusion, and truncation [11]. Our method allocates 1/3 of the 7481 samples from the original KITTI training set as the validation split (2483 samples), and the remaining 2/3 as the training split (4998 samples) to facilitate model training. For every point cloud frame associated with the training split, our method initially applies statistical filtering to eliminate outlier noise points, subsequently employing the designed SFGTS for data augmentation. Concerning the point cloud objects synthesized by the simulated LiDAR, the process involves randomly discarding certain points and introducing minor Gaussian perturbations drawn from N(0,0.5) to closely emulate real-world conditions, and the objects are rotated by a slight angle uniformly drawn from [−π/10, π/10] and randomly positioned within the scene.

### 5.2. Engaged Models

According to the research, it selectively concentrates efforts on four marvelous LiDAR-based models as references: PointPillars [5], PointRCNN [6], PVRCNN [7], and Part-A^2^ [55]. PointPillars [5] functions as the baseline model to underscore our enhancements. Additionally, given that FGSM [9] introduces perturbations at the point level, our study opted for PointRCNN [6], which leverages point feature extraction to sustain the examination of its potential adversarial vulnerabilities. Furthermore, PVRCNN [7] is also selected, as it amalgamates point and voxel features to explore the influence of perturbations on voxel representations. Finally, Part-A^2^ [55] is included as well, owing to its deeper architecture compared to the above three. These four models are primarily employed to investigate the impact of grid-based and point-based representations on the enhancement of model performance in our approach. Moreover, in light of the remarkable performance exhibited by the recent transformer module, diffusion model, and graph convolution modules across various domains [56], our research also selects three state-of-the-art 3D object detectors, i.e., OcTr [57], SAFDNet [58], and Graph RCNN [59]; each one is constructed based on the former three frameworks, respectively. During adversarial training and adversarial data generation, our method entrusts PointNet [21] and Cylinder3D [53] as the attacker models. PointNet [21] undergoes pre-training on a curated ground truth dataset (generated from mesh data and KITTI dataset [11]), while Cylinder3D [53] pre-trains on the SemanticKITTI dataset [60].

### 5.3. Experimental Settings

#### 5.3.1. Basic Settings

Unless explicitly stated in the experimental study, during adversarial training, our method utilized the Adam optimizer with an initial learning rate of $2\times 10^{-4}$. The learning rate decreased by a factor of 0.8 for every 20 epochs, culminating in a total of 100 epochs, with a batch size configured to 2. The shape parameters (C, H, and W of the generated pseudo-image) were set to 64, 496, and 432 initially; the convolutional kernel size was set to K∈{3,5}, and the stride was S∈{1,2}. Each pillar grid was configured with dimensions of 0.24 m × 0.24 m in the horizontal direction and a height of 4 m. The maximum count of pillars was set to 12,000, with a maximum per-pillar point count of 100. For non-maximum suppression (NMS), the 2D intersection over union (IoU) threshold for the car class was set to 0.7, while for the pedestrian and cyclist classes, it was set at 0.5. For cars, anchor boxes with IoU ≥ 0.7 were classified as positive samples, whereas those with IoU < 0.5 were designated as negative ones. For pedestrians and bicycles, the anchor boxes exhibiting IoU ≥ 0.5 were regarded as positive instances, while those with IoU < 0.5 were aligned with negative ones. All measurements were conducted on GeForce RTX 4090 GPUs, utilizing a software environment comprising Ubuntu 22.04, Python 3.8.0, PyTorch 1.9.0, and CUDA 11.1.

#### 5.3.2. Settings on Hyperparameters *k* and $\gamma_{min}$

**Figure 12:**
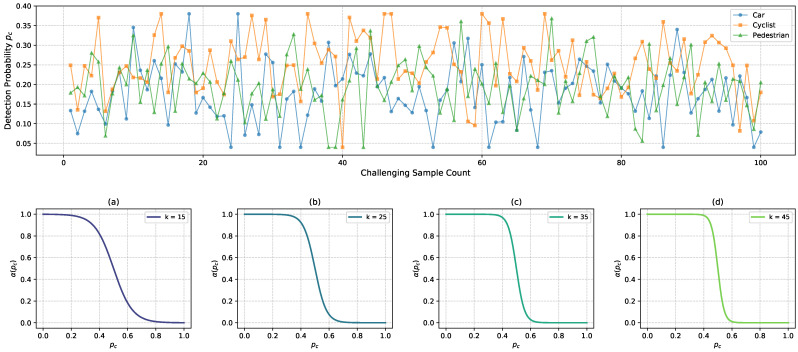
Detection confidence $p_{c}$ of three categories of difficult samples (top line) and the relationship between α and $p_{c}$ at different *k* values (bottom line). The challenging samples for cars (fewer than 30 points), cyclists and pedestrians (fewer than 20 points) originate from the Adv-KITTI dataset. The detection probabilities of these challenging samples are generally below 0.4, and as shown in diagrams (**a**–**d**), the weight boundaries between challenging and simple samples become distinct as *k* increases.

As illustrated in the top row in Figure 12, by randomly selecting 50 challenging samples for cars, cyclists, and pedestrians from Adv-KITTI, specifically targeting those with fewer than 30 points for cars and fewer than 20 points for cyclists and pedestrians, our research finds that the recognition probabilities, $p_{c}$, of these samples ranged from 0.04 to 0.38 by employing PointPillars [5] for detection. In order to enhance the model’s attention on these challenging samples, as depicted in the bottom row in Figure 12, diagrams (a) to (d), the curve progressively covers the range of $p_{c}$ from 0 to 0.4 as *k* increases gradually from 15 to 45. As for the $\gamma_{min}$ mentioned in Section 4.5, the original paper, Ref. [5], fixed $\gamma_{min}$ at 2, which fails to dynamically adapt to the difficulty of the samples. Consequently, our research conducts a grid search on these two hyperparameters by decreasing *k* from the initial value of 45 in increments of 5 in both upward and downward directions, and adjusting $\gamma_{min}$ from 2 in steps of 0.5 in both directions, which is implemented by our method based on Pillar-RBFN. The results of this search are presented in Table 2, where it can be seen that the optimal outcome is achieved when k=40 and $\gamma_{min}=1.5$, and our method ultimately designates this configuration.

### 5.4. Implementation Details

#### 5.4.1. Hierarchical Sample Partition

This research devises a purposeful experiment to assess the effectiveness of structuring data in accordance with a curriculum. Specifically, it preemptively organizes the training split in ascending order of difficulty through the methodology described in Section 4.1. In addition, the KITTI dataset [11] exhibits a partial class imbalance, as shown in Figure 5, with the car category overwhelmingly dominant; thus, the single-frame ground truth sample (SFGTS) [24] implemented in Algorithm 1 is used to augment each point example by slotting plentiful novel objects. It can be observed that the counts of the rarer categories—cyclist and pedestrian—have nearly reached the upper limit of the car category, after SFGTS [24]. In order to derive the precise item acc(P), i.e., mean average precision (mAP), our method trains a PointPillars [5] model on the nuScenes dataset beforehand [52], where the mAP for each frame in the training split is applied to guide the partitioning process. The weight factor α is set to 0.2, while both β and γ are equivalently assigned to 0.4. Compared to the KITTI dataset [11], the nuScenes dataset [52] provides a more comprehensive array of urban driving scenario data. Our method leverages this pre-trained model, considering it well-equipped to offer valuable guidance. Subsequently, the organized training split is fed to train the seven LiDAR-based models mentioned above, as well as ours, while concurrently training an additional five identical models with the unorganized training split for comparative analysis. It is noteworthy that Pillar-RBFN utilizes the identical loss function employed by the original PointPillars [5] during the training process.

#### 5.4.2. PointPillars with RBFN

Before adversarial training, an unadorned experiment is conducted to affirm the judiciousness of employing the nonlinear enhancement block (NEB). Specifically, the perturbation method outlined in Section 4.2 is preliminarily applied to the original training split that transitions through the hierarchical sample partition (HSP). For foreground points (i.e., belonging to positive and GT examples) and background points, PointNet [21] and Cylinder3D [53] are employed to implement adversarial perturbations, respectively. PointNet [21] is pre-trained on the curated ground truth dataset (generated from mesh data and the KITTI dataset [11]), while Cylinder3D [53] is pre-trained on the SemanticKITTI dataset [60]. Notably, the state ϵ=0 corresponds to standard training without inflicting perturbations. Subsequently, the five models are evaluated for adversarial robustness using the perturbed training split.

#### 5.4.3. Adversarial Training

**Figure 13:**
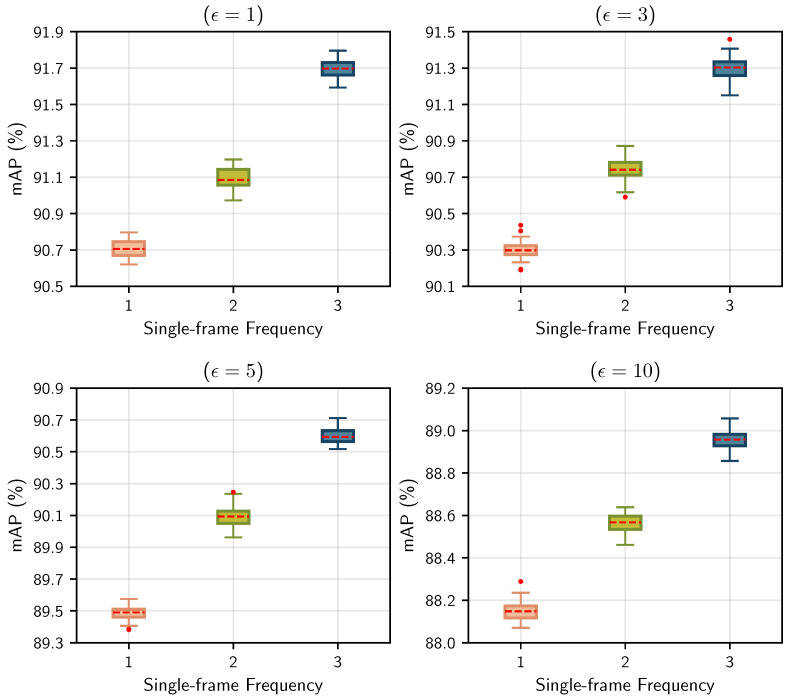
An illustration of the relationship between the mean average precision (mAP) and the number of reviews of the current frame on 50 models with different initialized weights during adversarial training. The red points represent outliers, while the red dashed line indicates the median value. It is evident that even with intensified perturbation magnitudes, ϵ∈{1,3,5,10}, increasing the model’s review count of the current point cloud frame can improve its mAP by approximately 1 percentage point, which reveals that under varying levels of perturbation, a judicious setting in repetition frequency can benefit the training process.

Following the arrangement of the training split, our method implements the three-stage strategy delineated in Section 4.4 to train Pillar-RBFN. In the first stage, it engages in sequential learning from the curriculum training split; the NEB module remains frozen and, thus, the model parallels the PointPillars [5], with the category optimization objective articulated in Equation (18). This research initially set k=40 and $\gamma_{min}=1.5$, which has been analyzed above. Figure 11, diagram (c), illustrates the relationship between α and the number of epochs, *e*, during a specific training cycle of a car object, while diagram (d) depicts the variations of γ with respect to *e*. It can be seen that during the early phases of training (0≤e<28), objects with lower $p_{c}$ receive elevated weights, followed by a rapid decline in weight α over a few epochs (28≤e<49), ultimately reaching a wavering state (49≤e≤100). This indicates that our designed loss function effectively directs the model’s attention toward challenging objects. In the second stage, the NEB module and adversarial sample generation system are activated. After one single iteration, each frame serves to synthesize adversarial counterparts. For the foreground and background points, our method commissions the attacker models—PointNet [21] and Cylinder3D [53]—to simulate black-box perturbation attacks. During this process, our method explores the following two configuration approaches for the perturbation magnitude: fixed settings and adaptive settings of ϵ. For the fixed manner, several options of ϵ are established: ϵ∈{0.5,1,2,3,5,8,10}. For the adaptive manner, the parameter ϵ in Equation (9) is initially set to the value mentioned above, allowing it to be updated during the adversarial training as described in Section 4.2. In the third stage, the modules associated with PointPillars [5] are frozen, granting the NEB module exclusive access to adversarial data. It merits attention that each stage tends to propagate the current frame’s three iterations, while the second stage alternates between inputting the training frames and adversarial frames three times. Such handling mirrors human learning, where individuals typically review a given problem multiple times. Figure 13 illustrates the relationship between the mean average precision (mAP) of the Pillar-RBFN during adversarial training and the repetition count of the current frame per epoch, which reveals that under varying levels of perturbation, a judicious increase in repetition frequency significantly enhances accuracy performance. To investigate the efficacy of our adversarial training strategy, our method conducts regular adversarial training on another Pillar-RBFN by sequentially inputting curriculum samples alongside the corresponding adversarial examples.

### 5.5. Experimental Results

#### 5.5.1. Curriculum Partitioning Analysis

Table 3 presents a comparison of the 3D object detectors mentioned above, trained on both the curriculum training split and the randomly shuffled training split, respectively. It can be seen that all eight detectors, including our method, achieve improvements in detection accuracy across all three main categories after curriculum arrangement, with the mAP showing an improvement range of approximately 0.5∼0.8 percentage points. These results underscore that a meticulously designed input process based on curriculum principles can guide the model’s learning in favorable directions. Table 4 illustrates the comparative results of detectors subjected to SFGTS versus HSP (i.e., SFGTS + curriculum arrangement). It is noteworthy that after utilizing the augmented dataset, i.e., Adv-KITTI, all the detectors can obtain comprehensive enhancement of at least 15 percentage points in mAP across three categories, particularly with the relatively challenging category—pedestrian—achieving an extraordinary increase of at least 10 percentage points. This highlights the considerable advantages of effective data augmentation in enhancing model performance, particularly given that class imbalance can severely limit the model’s potential. Furthermore, the utilization of curriculum arrangement based on SFGTS (i.e., HSP) stimulates the mAP to reach its peak with an enhancement of 0.9∼1.5 percentage points. This insinuates the tremendous potential of HSP as a data augmentation strategy, and it further certifies that the relative process related to curriculum principles can be trusted as an auxiliary measure of data augmentation to additionally elevate performance.

#### 5.5.2. Quantitative Adversarial Robustness Evaluation

**Figure 14:**
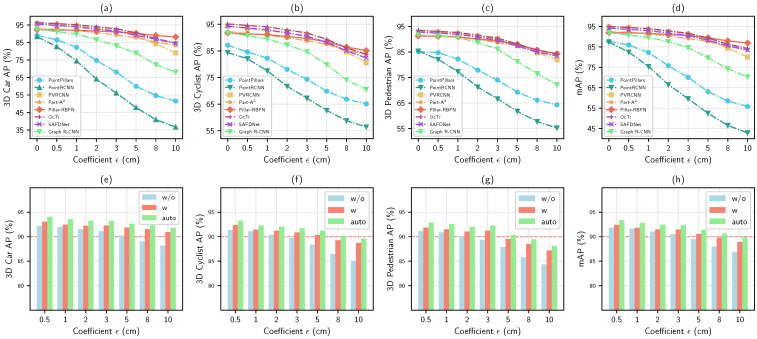
The adversarial robustness evaluation of several LiDAR-based detectors on the evaluation split with different perturbation magnitudes ϵ∈{0.5,1,2,3,5,8,10}, where the figure (**a**–**d**) exclusively utilize HSP in Section 4.1 for normal training. ‘w/o’ signifies the absence of our proposed adversarial training strategy in the Pillar-RBFN, ‘w’ denotes its implementation with the fixed ϵ, and ‘auto’ indicates the auto-updation of it in the figure (**e**–**h**).

Figure 14, diagrams (a–d), present a quantitative adversarial robustness evaluation of the seven 3D object detectors, and diagrams (e–h) dedicate the evaluation of different manners of adversarial learning on Pillar-RBFN, where ‘w/o’ signifies the absence of the proposed adversarial training strategy; ‘w’ denotes its implementation with the fixed ϵ; and `auto’ indicates the auto-updation of it.

- Analysis on different feature representations.

The results in Figure 14, diagrams (a–d), apparently indicate that, in the absence of adversarial training, Pillar-RBFN remains more unbending (i.e., exceeding 85% in mAP) relative to its four peers, i.e., PointPillars [5], PointRCNN [6], PVRCNN [7], and Part-A^2^ [55]. Compared to the original PointPillars [5], the incorporation of the nonlinear enhancement block (NEB) yields an increase of over 4.6 percentage points in mAP for undisturbed conditions, ϵ=0. Conversely, the original PointPillars [5] exposes a declining trend as perturbations increase. It indicates that our construction, with the NEB module, substantially enhances the adversarial robustness of PointPillars [5]. Moreover, at the initial state without perturbation, the accuracy of Pillar-RBFN across three categories is slightly inferior to those of Part-A^2^ [55] and PVRCNN [7] (with mAPs of 92.32%, 92.96%, and 95.86%, respectively). However, as perturbations intensify, the latter two gradually lag behind (with an mAP of 88.18% for Pillar-RBFN, 83.39% for Part-A^2^ [55], and 79.07% for PVRCNN [7] at ϵ=10), which further demonstrates the inherent advantage of the module NEB in resisting adversarial perturbations. In contrast to PVRCNN [7], which integrates both point and voxel features, the point-based method PointRCNN [6] exhibits heightened vulnerability to perturbations, with its mAP declining from an initial percentage of 86.36% to a final percentage of 49.45%. This observation underscores the superior adversarial robustness of voxel feature representation when confronting perturbations compared to point feature representation. Ultimately, Part-A^2^ [55] also demonstrates competitive adversarial robustness, indicating that the conclusion regarding the enhancement of adversarial resilience through nonlinearity is similarly applicable within the 3D domain.

- Analysis on three state-of-the-art modules.

Our research explores three other special 3D object detectors, i.e., OcTr [57], SAFDNet [58], and Graph RCNN [59], which are constructed based on three state-of-the-art modules, i.e., transformer, the diffusion model, and the graph convolution module, respectively. It can be observed in Figure 14, diagrams (a–d), that as the perturbations intensify, when ϵ equals 10, the mAPs of OcTr [57] and SAFDNet [58] (84.23% and 83.45%, respectively) approach Part-A^2^ [55], slightly surpassing the PVRCNN [7]. In contrast, the mAP of Graph RCNN [59] (70.29%) is inferior to PVRCNN [7], yet it remains much higher than both PointPillars [5] and PointRCNN [6], with increments of 14.49% and 27.41%, respectively. It can be inferred that, for the former two methods, the multi-head mechanism of the transformer may mitigate the impacts of adversarial perturbations, alleviating their propagation within deep networks, and the stochastic sampling process of the diffusion model similarly dilutes the effects of deterministic attacks. Nevertheless, they remain incapable of fully defending against targeted perturbations. In the case of Graph RCNN [59], the multi-layer aggregation of graph convolutions may cause node features to converge, allowing adversarial perturbations to be amplified through multi-layer propagation, ultimately leading to a decline in detection accuracy. Overall, Pillar-RBFN exhibits suboptimal accuracy initially, whereas its performance curve progressively surpasses the other three models as the perturbation magnitude intensifies to a level of 5. This indicates that the NEB module demonstrates superior adversarial robustness compared to the previously mentioned three modules.

- Analysis on different setting manners.

After pertinent adversarial training, it becomes apparent from Figure 14, diagrams (e–h), that Pillar-RBFN gains further strength. The light blue bars represent outcomes derived from regular adversarial training, the light red bars illustrate the efficacy of our tailored adversarial training strategy with fixed ϵ settings, and the light green bars denote that ϵ is updated automatically. Once the perturbation magnitude ϵ surpasses 5, regular adversarial training causes the average precision (AP) of all three categories to drop below 90% and drastically decline as the perturbation increases, whereas the fixed and adaptive approaches maintain performance at the baseline level, indicating that our training strategy demonstrates superior effectiveness in guiding the model to withstand perturbation attacks. In a comprehensive evaluation, the method of automating the update of the perturbation magnitude significantly enhances the average precision (AP) by approximately 0.8 to 1.1 percentage points across all three categories, achieving an average increase of at least 0.9 percentage points in mean average precision (mAP). This indicates that a fixed parameter setting for ϵ may result in the model overlooking superior local optima, whereas the automated updating approach can circumvent this blindness to yield a more robust model.

#### 5.5.3. Qualitative Adversarial Robustness Evaluation

**Figure 15:**
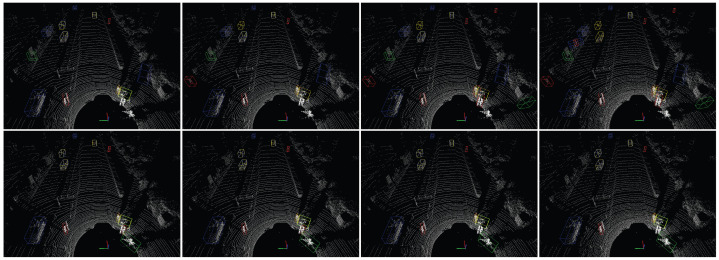
The 3D visualization of the detection results of Pillar-RBFN (**bottom row**) and the original PointPillars [5] (**top row**). The perturbation magnitudes ϵ are sequentially set to 0, 1, 5, and 10 from the left to right. The red boxes, green boxes, and blue boxes, respectively, frame the predicted pedestrians, cyclists, and cars, while the yellow box indicates the ground truth. It can be observed that both Pillar-RBFN and PointPillars exhibit good generalization capabilities. However, as the perturbation magnitude intensifies, PointPillars gradually predicts more false positives and false negatives, while Pillar-RBFN remains relatively stable.

Figure 15 visualizes the comparative detection results of Pillar-RBFN and the original PointPillars [5] under different perturbation magnitudes (the ϵ values are sequentially set to 0, 1, 5, and 10). In the absence of perturbations at ϵ=0, PointPillars [5] exhibits a notable proficiency in predicting the positions of ground truth (GT) elements, while simultaneously demonstrating a commendable level of generalization capability. Nevertheless, it continues to predict a certain number of false positives and false negatives. As perturbations escalate, the model progressively fails to rationally predict the positions of GT elements, resulting in the emergence of more abundant false positive objects. In particular, when ϵ=10, it catastrophically predicts eight false positives (comprising three cars, three pedestrians, and two cyclists). Concurrently, the 3D bounding boxes produced by our model not only achieve a higher degree of accuracy in aligning with the positions of the GT boxes but also demonstrate superior generalization by identifying two objects overlooked by PointPillars (one pedestrian and one cyclist). Moreover, as perturbations increase, our model avoids the phenomenon of ground truth predictions deviating from their bounding boxes and does not generate an abundance of false positive objects. Only at the state of ϵ=10 does it fail to generalize one existing pedestrian.

### 5.6. Other Relative Experiments

#### 5.6.1. Adversarial Robustness with Different Pillar Sizes

In order to elucidate the impact of pillar dimensions on the adversarial robustness of the models, our method undertakes a series of comparative experiments, employing five distinct pillar sizes, focusing on both the original PointPillars [5] and Pillar-RBFN. Table 5 illustrates the impact results of varying pillar sizes. It is apparent that increasing the size of the pillars across varying perturbation amplitudes yields oscillatory results in the mean average precision (mAP) for both PointPillars [5] and Pillar-RBFN, with a discernible overall trend toward declining. In particular, at ϵ=10, modifications to the pillar sizes provide negligible benefits to the performance of PointPillars [5]; thus, one can conclude that the pillar size is a secondary factor that influences the adversarial robustness of the model. It is worth mentioning that this procedure excludes the utilization of our hierarchical sample partition and adversarial training techniques.

#### 5.6.2. Ablative Study on Adversarial Robustness Enhancement

Table 6 delineates the impact of various modules on the adversarial robustness of Pillar-RBFN, with NEB representing the nonlinear enhancement block, HSP denoting the hierarchical sample partition mentioned in Section 4.1, and CRT signifying curriculum robustness training mentioned in Section 4.4. It can be observed that the original PointPillars [5] experiences a significant decline in mAP by 47.22 percentage points as ϵ escalates from 1 to 10 (row 1). After the incorporation of the NEB module (row 2, which denotes Pillar-RBFN), it not only exhibits improved detection accuracy across various perturbations (vertical line), but also demonstrates a mere decrement of 3.51 percentage points as perturbation elevates (horizontal line), indicating that the inclusion of NEB can substantially enhance the adversarial robustness of PointPillars [5]. Following the further integration of the HSP module (row 3), the mAP of PointPillars increases by at least 18 percentage points, demonstrating that HSP effectively enhances detection accuracy. Subsequent to additional training with CRT (row 5, which represents our proposed framework), the mAP experiences a modest enhancement, while concurrently decreasing by 2.84 percentage points as perturbation levels escalate. Moreover, after accounting for the potential influences of NEB and CRT (row 4), the mAP declined by 19.1 percentage points on average, suggesting that CRT further augments the model’s adversarial robustness. Table 7 explores the effects of splitting the CRT, specifically examining the individual impacts of curriculum Learning (CL) and adversarial training (AT) on the Pillar-RBFN. It can be observed that when both CL and AT are absent, the mAP of Pillar-RBFN decreases by 6.26 percentage points as the perturbation magnitude increases from 1 to 10. After introducing CL, the mAP of Pillar-RBFN improves by 1.40 percentage points across various perturbations yet subsequently declines by 6.34 percentage points as perturbations increase. Furthermore, the subsequent combination of AT results in an average increase of 1.54 percentage points in mAP across different perturbations, with a decline of approximately 4.75 percentage points as perturbations intensify. In terms of robustness enhancement, this indicates that adversarial training demonstrates a greater advantage compared to CL, while CL primarily contributes to improving accuracy. With respect to robustness enhancement, this underscores that adversarial training exhibits an advantage over CL, while CL predominantly serves to bolster the detection accuracy. Table 8 investigates the transferability of the proposed methods, focusing on the victim model PointRCNN [6], which is selected for its susceptibility to perturbations, as depicted in Figure 14. At a perturbation level of ϵ=5, it can be observed that the model becomes more demanding to recognize (row 1). The integration of NEB materially bolsters adversarial robustness (row 2) and can be further amplified through the application of CRT (row 3). Note that in PointRCNN [6], NEB is integrated at the forefront of the point cloud encoder.

## 6. Discussion

This section primarily discusses our method, beginning with an exploration of its potential practical applications and challenges, followed by an acknowledgment of its inherent limitations and a clarification of future research directions.

### 6.1. Potential Practical Applications and Challenges

The proposed framework employs a progressive approach to enhance adversarial intensity during model training, enabling the model to adapt to dynamic environments, such as the transition from light rain to heavy downpour in real-world scenarios. Furthermore, the NEB module can leverage the high nonlinearity of radial basis functions to fit the local geometric configurations of target point clouds, such as vehicle edges, thereby stabilizing detection accuracy and ensuring driving safety. Finally, the adaptive focal loss effectively directs the model’s attention toward small objects characterized by sparse point clouds, which can mitigate the risk of safety incidents akin to ghosting phenomena. However, our method still confronts several potential challenges. While the incorporation of the NEB module into the PointPillars can enhance the adversarial robustness, it may concurrently elevate computational latency, thereby presenting substantial challenges for deployment in resource-constrained environments. Moreover, the necessity of generating adversarial samples at multiple intensities during curriculum adversarial training increases the training time cost, which may present challenges for the centralized distribution model updates of perception models in large-scale fleets.

### 6.2. Limitations and Future Research Directions

In addition to the previously mentioned limitations related to training overheads and computational burdens, it can also be observed that our method exclusively investigates FGSM-based attacks, which may generate perturbations that exhibit fixed patterns and lack diversity. Additionally, during the GTS process, the randomly positioned target point clouds may result in illogical placements, such as overlapping between the objects and ground areas. Finally, our investigation solely addresses the adversarial robustness of LiDAR point cloud data, whereas cameras are also indispensable sensors in autonomous driving systems. Therefore, future research ought to explore more efficient adversarial training frameworks, such as those based on knowledge distillation or dynamic sparse training, to compress the curriculum adversarial framework for deployment on edge devices. Additionally, further refining the relevant algorithms to address their shortcomings and extending the framework to multi-modal adversarial training—incorporating both cameras and LiDAR—is required, thereby tackling the issue of sensor collaborative attacks.

## 7. Conclusions

This research introduces a robust enhancement training framework that markedly improves the resilience of PointPillars against adversarial perturbation attacks implemented by the incorporation of a nonlinear enhancement block. By implementing a novel generation method of adversarial perturbations, diverse adversarial examples are produced, which can be utilized to train the Pillar-RBFN adversarially. Moreover, it further proposes a novel point cloud augmentation method and an adaptive focal loss, which can tackle the class imbalance issue within the KITTI dataset effectively. The experiments conducted evidently demonstrate that the framework contributes to enhancing the detection accuracy and adversarial robustness of LiDAR-based models, demonstrating the potential for transferability to other detectors. 

## Figures and Tables

**Table 1:** A comparison between point-based and grid-based methods. PB denotes the point-based representation. GB indicates the grid-based representation. The memory footprint is categorized into three distinct levels: L, M, and H, where L denotes a range of 0∼20 mb, M represents a range of 20∼50 mb, and H indicates values exceeding 50 mb.

Method	Modality	Representation			Feature Extraction				Memory Footprint		
		Point	Voxel	Pillar	2D cnn	3d cnn	Transformer	Set Abstraction	L	M	H
PointRCNN [6]	PB	✔	-	-	-	-	-	✔	-	-	✔
3DSSD [19]	PB	✔	-	-	-	-	-	✔	-	-	✔
Pointformer [20]	PB	✔	-	-	-	-	✔	-	-	✔	-
PointPillars [5]	GB	-	-	✔	✔	-	-	-	✔	-	-
SECOND [24]	GB	-	✔	-	✔	✔	-	-	-	✔	-
VoxelNet [23]	GB	-	✔	-	-	-	-	✔	-	-	✔
VoTr-SSD [25]	GB	-	✔	-	✔	-	✔	-	-	✔	-

**Table 2:** Grid search of the hyperparameter *k* and $\gamma_{min}$ under different settings. Recall is recorded for the pedestrian category, which is relatively challenging to detect, and the epoch denotes the total count of iterations until convergence. The bolded one denotes the optimal.

*k*	$\gamma_{\min}$	mAP (%)	Recall (%)	Epoch
35	1.0	87.31	72.50	124
35	1.5	88.64	73.82	116
35	2.0	87.90	73.13	118
35	2.5	89.47	74.56	115
40	1.0	90.24	76.71	104
**40**	**1.5**	**91.97**	**78.90**	**101**
40	2.0	90.68	77.54	106
45	1.0	89.52	75.28	110
45	1.5	90.10	76.10	112
45	2.0	89.34	74.83	108
50	1.0	86.75	70.92	116

**Table 3:** Performance comparison on the KITTI validation split. RS: shuffle the training split randomly. CA: arrange the training split with the curriculum.

Method	Modality	mAP (%)		3D Car AP (%)		3D Cyclist AP (%)		3D Pedestrian AP (%)	
		RS	CA	RS	CA	RS	CA	RS	CA
PointPillars [5]	LiDAR	69.11	69.79	78.59	79.17	69.82	70.68	58.94	59.51
PointRCNN [6]	LiDAR	69.84	70.61	79.18	80.24	70.12	70.86	60.22	60.73
PVRCNN [7]	LiDAR	74.59	75.19	82.70	83.41	74.37	74.95	66.71	67.20
Part-A^2^ [55]	LiDAR	73.72	74.38	80.51	81.48	76.90	77.43	63.76	64.24
Graph R-CNN [59]	LiDAR	74.64	75.33	82.57	83.32	74.78	75.47	66.58	67.21
OcTr [57]	LiDAR	75.82	76.48	83.72	84.45	75.91	76.58	67.83	68.42
SAFDNet [58]	LiDAR	75.34	76.03	83.28	84.02	75.45	76.12	67.30	67.96
Pillar-RBFN (ours)	LiDAR	71.51	72.08	80.26	81.02	72.45	72.86	61.82	62.37

**Table 4:** Performance comparison on the KITTI validation split. GTS: single-frame ground truth sample, which can also be regarded as Adv-KITTI. HSP: hierarchical sample partition.

Method	Modality	mAP (%)		3D Car AP (%)		3D Cyclist AP (%)		3D Pedestrian AP (%)	
		GTS	HSP	GTS	HSP	GTS	HSP	GTS	HSP
PointPillars [5]	LiDAR	85.73	87.13	87.23	89.08	86.35	87.14	83.60	85.17
PointRCNN [6]	LiDAR	85.06	86.03	87.42	88.36	82.84	84.36	84.91	85.38
PVRCNN [7]	LiDAR	92.82	94.37	95.17	95.86	92.56	94.38	90.73	92.87
Part-A^2^ [55]	LiDAR	90.56	92.26	91.80	92.94	90.12	92.26	89.76	91.58
Graph R-CNN [59]	LiDAR	88.59	89.96	89.45	90.83	88.32	89.67	88.01	89.38
OcTr [57]	LiDAR	93.20	94.62	95.42	96.18	93.25	94.72	90.92	92.95
SAFDNet [58]	LiDAR	91.19	92.89	92.87	93.95	91.08	92.67	89.63	91.42
Pillar-RBFN (ours)	LiDAR	89.57	91.73	90.31	92.17	89.66	91.74	88.58	91.28

**Table 5:** The impact results of varying pillar sizes on adversarial robustness under different magnitudes of perturbation. The mAP (%) is recorded.

	PointPillars [5] (%)			Ours (%)		
	ϵ=1	ϵ=5	ϵ=10	ϵ=1	ϵ=5	ϵ=10
0.18 m	64.73	36.29	8.20	73.18	72.93	70.46
0.20 m	61.32	30.42	11.34	73.60	72.11	69.27
0.22 m	59.26	34.15	5.71	72.64	71.14	68.80
0.24 m	62.12	27.94	14.90	72.05	70.83	68.54
0.26 m	58.40	22.03	10.57	72.34	71.62	66.82

**Table 6:** Ablative study of different functional modules on enhancing adversarial robustness. The red color indicates missing modules, while green indicates engaged modules. NEB: nonlinear enhancement block. HSP: hierarchical sample partition. CRT: curriculum adversarial training.

NEB	HSP	CRT	mAP (%)		
			ϵ=1	ϵ=5	ϵ=10
✘	✘	✘	62.12	27.94	14.90
✔	✘	✘	72.05	70.83	68.54
✔	✔	✘	91.65	89.49	86.90
✔	✘	✔	73.52	71.73	68.75
✔	✔	✔	91.80	90.58	88.96

**Table 7:** Ablative study of curriculum learning (CL) and adversarial training (AT) on Pillar-RBFN, separated from the curriculum adversarial learning (CRT). The red color indicates missing modules, while green indicates engaged modules.

CL	AT	mAP (%)		
		ϵ=1	ϵ=5	ϵ=10
✘	✘	71.22	68.94	64.96
✔	✘	72.69	70.30	66.35
✔	✔	73.47	71.79	68.72

**Table 8:** Ablative study on adversarial robustness enhancement of the nonlinear enhancement block (NEB) and curriculum adversarial training (CRT) across other 3D object detectors. The red color indicates missing modules, while green indicates engaged modules.

NEB	CRT	mAP (%)
		ϵ=5
✘	✘	18.52
✔	✘	69.76
✔	✔	71.10

## Data Availability

The raw data supporting the conclusions of this article will be made available by the authors upon request.
